# Characterisation of Peptide Microarrays for Studying Antibody-Antigen Binding Using Surface Plasmon Resonance Imagery

**DOI:** 10.1371/journal.pone.0012152

**Published:** 2010-08-13

**Authors:** Claude Nogues, Hervé Leh, Christopher G. Langendorf, Ruby H. P. Law, Ashley M. Buckle, Malcolm Buckle

**Affiliations:** 1 Dynamics of Macromolecular Complexes, Laboratoire de Biologie et Pharmacologie Appliquée, UMR 8113 du CNRS, Institut d'Alembert, Ecole Normale Supérieure de Cachan, Cachan, France; 2 Department of Biochemistry and Molecular Biology, Monash University, Clayton, Victoria, Australia; University of Maryland-Baltimore, School of Pharmacy, United States of America

## Abstract

**Background:**

Non-specific binding to biosensor surfaces is a major obstacle to quantitative analysis of selective retention of analytes at immobilized target molecules. Although a range of chemical antifouling monolayers has been developed to address this problem, many macromolecular interactions still remain refractory to analysis due to the prevalent high degree of non-specific binding. We describe how we use the dynamic process of the formation of self assembling monolayers and optimise physical and chemical properties thus reducing considerably non-specific binding and allowing analysis of specific binding of analytes to immobilized target molecules.

**Methodology/Principal Findings:**

We illustrate this approach by the production of specific protein arrays for the analysis of interactions between the 65kDa isoform of human glutamate decarboxylase (GAD65) and a human monoclonal antibody. Our data illustrate that we have effectively eliminated non-specific interactions with the surface containing the immobilised GAD65 molecules. The findings have several implications. First, this approach obviates the dubious process of background subtraction and gives access to more accurate kinetic and equilibrium values that are no longer contaminated by multiphase non-specific binding. Second, an enhanced signal to noise ratio increases not only the sensitivity but also confidence in the use of SPR to generate kinetic constants that may then be inserted into van't Hoff type analyses to provide comparative ΔG, ΔS and ΔH values, making this an efficient, rapid and competitive alternative to ITC measurements used in drug and macromolecular-interaction mechanistic studies. Third, the accuracy of the measurements allows the application of more intricate interaction models than simple Langmuir monophasic binding.

**Conclusions:**

The detection and measurement of antibody binding by the type 1 diabetes autoantigen GAD65 represents an example of an antibody-antigen interaction where good structural, mechanistic and immunological data are available. Using SPRi we were able to characterise the kinetics of the interaction in greater detail than ELISA/RIA methods. Furthermore, our data indicate that SPRi is well suited to a multiplexed immunoassay using GAD65 proteins, and may be applicable to other biomarkers.

## Introduction

Surface plasmon resonance imagery (SPRi) [1], [2] is a label free technique that avoids the use of fluorescence or radioactive labelling and offers a comparable dynamic range of detection, as well as access to kinetic constants not obtained by end point assays such as Radioimmunoassay (RIA). Furthermore it is a truly multiplexed assay, allowing the detection and measurement of ligand binding using a wide-range of immobilised target molecules simultaneously and in real-time. Performing a large amount of assays concurrently on one sensor surface offers a clear solution to problems of variability [3]. Finally, the application of micro fluidics opens up the exciting possibility of performing biosensor-based immunoassays using a range of antigens and tens-to hundreds of antibody/serum samples on one single chip.

A major stumbling block to the goal of achieving high throughput, rapid, quantitative analysis of peptide microarrays using SPRi is the difficulty of eliminating non-specific interactions with the surface containing the microarray target molecules. We have devised a novel surface chemistry (Nogues *et al.*, submitted) in which target macromolecules are immobilised at the metallic interface with the SPRi device in such a way that non-specific interactions between solution bound macromolecules and the surface are essentially non existent. This approach, optimised for protein-nucleic acid interactions was thus applied here to study the interaction between the 65kDa isoform of glutamic acid decarboxylase (GAD65) and a monoclonal antibody.

GAD65, but not GAD67, is a major autoantigen and autoantibodies to GAD65 are detected at high frequency in patients with newly diagnosed type 1 diabetes (T1D) (∼80%) [4]. Autoantibodies to GAD65 are considered an important diagnostic marker and are highly effective for predicting the development of T1D [5]. We reasoned that studying the interaction between GAD65 and monoclonal antibodies using SPRi might offer several insights. First, the recent crystal structure determination of both GAD65 and GAD67 [6] allowed existing epitope mapping studies [7], [8], [9] to be placed in a molecular context [7], [10], [11]. Structural knowledge of GAD65 also provides a higher level of control over its immobilisation on the biosensor surface. We are thus now in a position to probe the precise location and composition of these epitope sites. Second, establishing the binding kinetics of the GAD65-antibody interaction may pave the way for the development of a SPRi-based, high-throughput immunoassay for T1D. Measurement of autoantibodies against islet beta cell antigens is performed in many laboratories throughout the world, and repeated testing for combinations of β-cell autoantibodies is a major component of studies examining the progression to diabetes. At present such immunoassays (typically RIA and ELISA) are costly and labour intensive, as they are performed individually, antigen by antigen. However a single test for a diabetes-associated autoantibody to a single autoantigen cannot form the basis of an important clinical decision. Therefore screening for these autoantibodies should be performed using a range of antigens during different times in the disease process, and for disease prediction may have to be performed at distinct ages in order to achieve maximal predictive sensitivity and specificity. As such there is a pressing need for the development of novel validated immunoassays that are rapid, cheap, and high-throughput.

A multiplexed SPRi-based optical biosensor assay potentially offers many advantages over RIA and ELISA and has the potential to realize the high-throughput screening described above. We therefore decided to investigate the interaction between GAD65 and antibody using SPRi.

## Methods

### Preparation of protein chips

Protein chips were prepared on a glass prism (high refractive index n 1.7) activated by Reactive Ion Etching (RIE) prior to the thermal evaporation of a 50 nm gold layer as described in Nogues *et al.*, (submitted). A mixed self assembled monolayer of two alkanethiolates ^−^S(CH_2_)_11_(OCH_2_CH_2_)_4_OH, and ^−^S(CH_2_)_11_(OCH_2_CH_2_)_6_COOH was adsorbed on the gold surface as described previously [12]. Prior to protein deposition the prisms were thoroughly rinsed in pure ethanol for 20 min. The thiol coupling surface was prepared using a thiol coupling kit from Biacore following the standard Biacore protocol; the carboxylated surface was activated by incubation with a mixture of 1-ethyl-3-(3-dimethylaminopropyl) carbodiimide hydrochloride (EDC) (final concentration = 200 mM), N-hydroxysuccinimide (NHS) (final concentration = 50 nM) for 5 min at room temperature. A second incubation with 2-(2-pyridinyldithio) ethaneamine hydrochloride (PDEA) (final concentration = 175 mM) for 5 min at room temperature produced a thiol activated surface. Un-reacted activated carboxyl groups on the surface were blocked by incubation with ethanolamine (1M at pH 8.5) for 10 minutes.

The GAD65 protein was dialysed against phosphate buffered saline (PBS) in order to remove mercaptoethanol in the storage buffer and aliquots (100nL) at a final concentration of 0.5 mg/ml GAD65 were then deposited onto the fresh pre-treated prism surface using the Hamilton starlet robot and a modified pin tool protocol that minimised contact of the pin tool with the gold coated surface. Following GAD65 immobilization on the pre-treated surface via the covalent coupling of the thiol groups of the protein to the carboxylated-terminal of the surface, un-reacted groups on the surface were blocked as described in [13]. The prism was left for two minutes at room temperature in a sealed Petri dish at 100% relative humidity to prevent the spots from drying. Moreover, in order to reduce evaporation during the incubation, the protein solution contained 5% glycerol. The prism was then directly inserted into the SPRi apparatus and PBS buffer was immediately flowed across the surface at 25 µl/min.

### Preparation of GAD65 and mutants

Details of the expression and purification of recombinant human GAD65 have been published previously [6]. We used an N-terminally truncated form of GAD65 that lacked the first 89 residues; the N-terminal truncation facilitated purification because this region is hydrophobic and highly susceptible to proteolysis, and did not affect enzymatic properties [6] nor reactivity with specific antisera [14], [15], [16]. The proteins were expressed in *Saccharomyces cerevisiae* as fusions to a C-terminal hexahistidine tag, and purified from the cell lysate by immobilized metal affinity chromatography and size exclusion chromatography in the presence of glutamate and pyridoxal-5′-phosphate (PLP).

### Preparation of the monoclonal antibody GAD1

The mouse mAb GAD1, prepared from a BALB/c mouse immunized with partially purified chicken brain GAD [17] was a gift from M. Rowley (Monash University).

### SPR imaging setup

The SPRi machine was purchased from GenOptics. The biological interface consists of a prism surface coated with a thin layer (∼50nm) of gold. An evanescent field referred to as a plasmon wave is created at the interface of this gold-coated surface and the dielectric from a light beam when the light beam arrives at the interface at an angle of total internal reflection (TIR). At TIR there is a resonance effect, leading to a decrease in reflectance at a given angle. This is measured by imaging the entire reflected light from a monochromatic polarized electroluminescent diode using a camera linked via a dedicated optical system. Thus the whole surface of the imaged field containing many discrete spots with immobilised ligands may be analysed simultaneously. A microcuvette system allows material to be flowed across the surface and the SPR response at predetermined spots can be assessed in parallel by a time resolved CCD that captures changes in percentage reflectivity at selected spots on the surface. These changes, averaged across the surface of each spot as a function of time, are related to changes in concentration of mass at each spot, and thus provide access to the kinetics of interactions at the surface at each immobilised ligand. A characteristic of this technique compared to other SPR based devices is that non-specific interactions of the molecules directly with the surface around selected spots can be simultaneously quantified and compared with specific interactions occurring with the target material in the spots, assuming that the amount of non specific interaction inside and outside the spots are identical. A recent study has shown that kinetic constants extracted from the kinetic curves collected with a Biacore SPR apparatus or from the SPRi apparatus are comparable when using identical surfaces and conditions [2].

### Interactions with anti-GAD antibodies

GAD1 antibody (200 µl at various concentrations) was flowed across the immobilised GAD65 proteins in the SPRi apparatus at 20µl/ml at room temperature in PBS buffer.

### Data analysis

Prism software was used to fit curves and obtain apparent rate constants. Curves were fitted to a simple Langmuir binding model to obtain kinetic constants. The apparent dissociation rate constant k_off_ may be obtained by fitting the dissociation phase to a simple exponential expression where the relative change in resonance response (R) as a function of time (t) with respect to R at time t = 0, (R_(0)_), results from 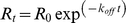 and the apparent association rate constant 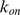 is obtained from 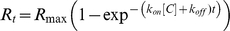 at a given protein concentration of 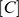, where 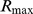 is the response at steady state. The calculated 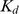 is simply the ratio of 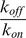. Three spots were analysed and the data represents the average of values obtained for these three spots.

## Results and Discussion

**Figure 1:**
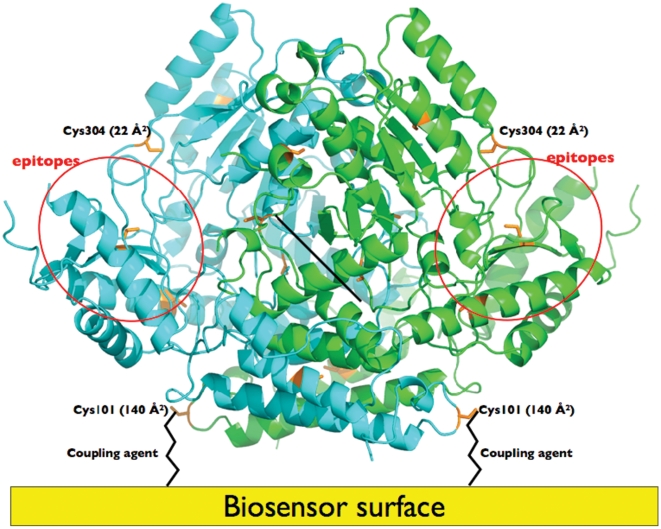
Structural characteristics of GAD65. Location of Cys residues (orange) on the GAD65 molecule. GAD65 molecules are coupled to the biosensor using Cys101 and coupling agent/linker. Domains are coloured separately. Putative epitope regions are indicated by red ellipses. Cysteine residues are indicated in orange, showing their accessible surface area (ASA), Cys101 being the only cysteine residue accessible for coupling to the biosensor surface. The PDEA coupling agent is indicated by black sticks.

**Figure 2:**
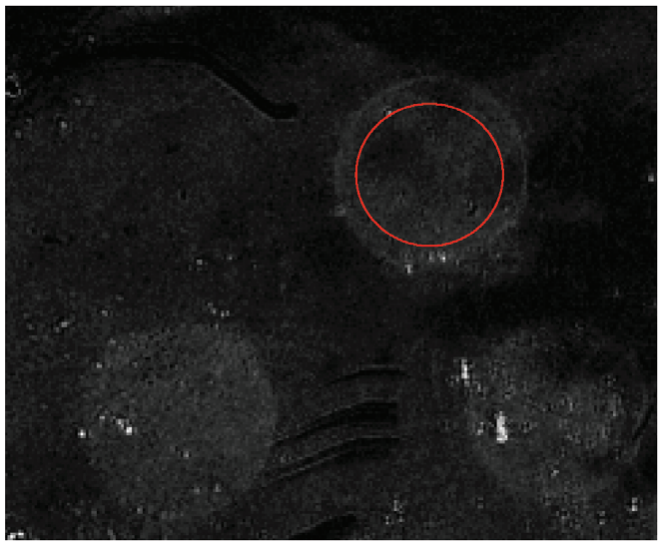
Difference images obtained from SPRi of antibodies binding to GAD65 immobilised at a gold/GLISS surface. Spots (400 µm diameter) containing GAD65 protein were immobilised by spotting at the surface using Hamilton Starlet equipped with a PinTool tip and modified software. GAD1 antibody (4.8 nM in 200 µl PBS) was flowed across the surface at 20 µl/min as described in Methods and differential images obtained using the GenOptics SPRi device. The red circle illustrates the area that is generally chosen to calculate the pixel density at any given time in order to generate curves of the type shown in Figure 3.

**Figure 3:**
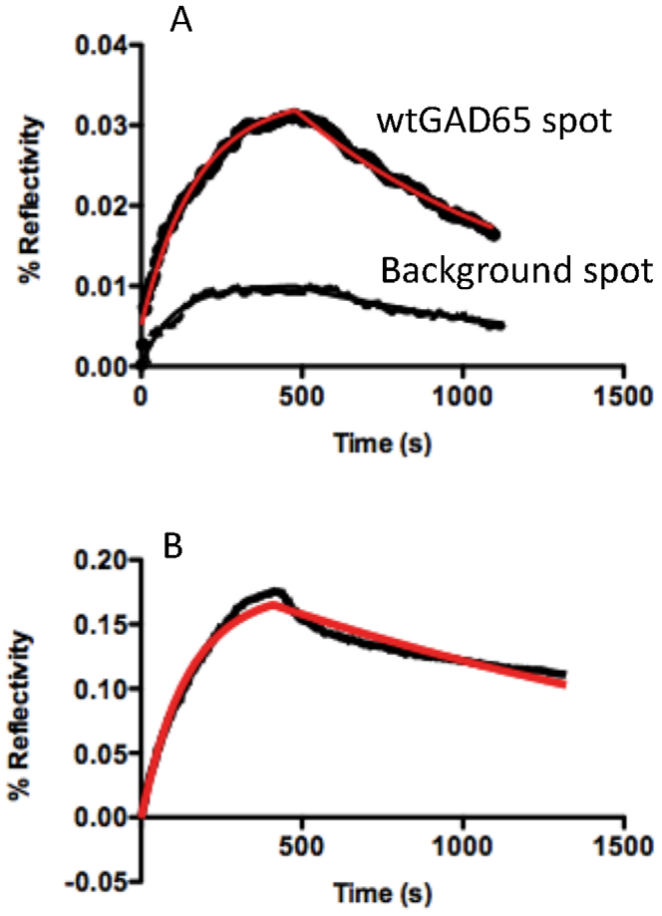
Changes in % reflectivity as a function of time as GAD1 antibody reacts with immobilised GAD65 protein. Curves were generated from calculating the change in % reflectivity in time across the spots of the sort shown in Figure 2. A) GAD1 antibody (4.8 nM) injected over the surfaces. The wtGAD65 curve was fitted to a simple binding model (red line) as described in Data Analysis to give the following dissociation (k_off_) and association (k_on_) values: k_off_ = 1.29±0.02·10^−3^ s^−1^; k_on_ = 9.41±0.29·10^5^ M^−1^s^−1^ from which an overall equilibrium dissociation constant, *K*_d_ of 1.37±0.06 nM, could be calculated. The background curve is taken from changes in surface pixel density at equivalent areas on the surfaces not containing immobilised GAD65; B) GAD1 antibody (48nM) injected over the surfaces. The association and dissociation phases gave a poor fit to a single binding site model and were clearly not monophasic.

The biochip surface that we have developed (Nogues *et al.*, submitted) consists of a 50 nm thick gold surface functionalised with hydroxyl-terminated tetra (ethylene glycol) (EG*4*-OH) to which the proteins had been linked via cysteine residue 101 on GAD65 (Figure 1). Typical images of the interaction of the antibody (4.8 nM) as it flows across the spots containing the immobilised GAD65 protein are shown in Figure 2. The images are differential images and are taken every two seconds during the experiment. The background is low and this is also evident from curves in Figure 3A showing the % reflectivity changes for the wild type protein GAD65 containing spot and background spot as antibody passes over the surface. At relatively low concentrations of the antibody (4.8 nM) a simple binding curve was observed (Figure 3A) from which apparent rate constants (shown in the legend to Figure 3) could be calculated. At these concentrations of antibody the protein GAD65-antibody interaction gave an overall apparent equilibrium dissociation constant (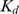) of 1.37 nM. We note that these values are somewhat different from values reported previously in the literature for this type of interaction [18]; we consistently found lower equilibrium dissociation constants essentially due to more rapid association rates that we measure here. There may be many explanations for this related to the difference in the immobilisation procedure, the architecture of the SPR machine, and so forth. However we were surprised to note that at higher concentrations of antibody (48nM) the binding phase became multiphasic or at least biphasic (Figure 3B). This observation was clearly not due to non-specific binding to the surface (Figure 3B shows a difference curve after subtraction of background), and indeed the observation of multiphasic binding is due precisely to the fact that we have very low non-specific binding to non target areas on the surface. We note that the concentrations of anti-GAD antibody used in the Biacore study of [18] were in excess of 500nM, which is 10 to a 100 fold in excess of the values that we use in the current manuscript and that this strongly suggests that the values obtained in [18] were somehow influenced by a degree of non specific binding, if not necessarily to the surface then perhaps to an abnormal form of the immobilised GAD.

**Figure 4:**
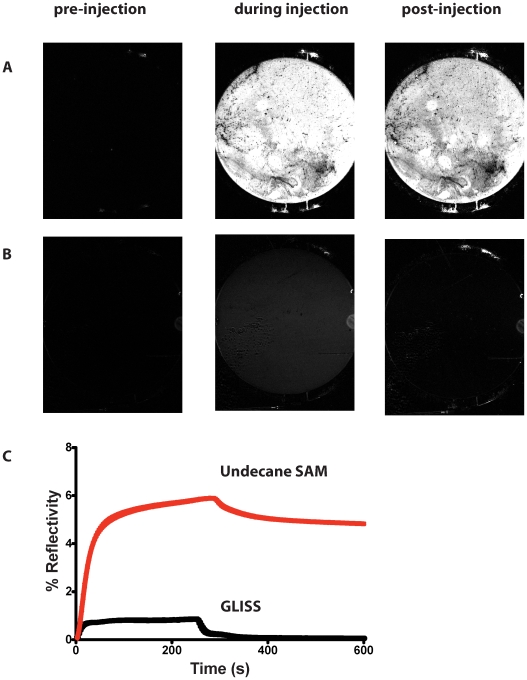
Differential binding to SPRi surfaces. Two independent surfaces were created either using classical SAM constructed from undecanoic acid as described in [2], or the GLISS protocol described here. Serum (stock concentration 60mg/ml diluted to a final concentration of 0.6 mg/ml) in PBS was passed across the surfaces at 25 µl/min. The three images for each surface refer to images prior to, during and after injection of the serum. A) Differential images of classical undecanoic based SAM surfaces B) Differential images of GLISS prepared surfaces C) Changes in % reflectivity as a function of time derived from images of the type shown in A and B.

In order to illustrate the degree of antifouling conferred by the General Liquid Interface Specific Surfaces (GLISS) we in fact passed human serum across two types of surface, one consisting of a classical self assembling monolayer (SAM) constructed from undecanoic acid as described in [2], and the other using the GLISS surfaces used in the current GAD65 study. As can be seen in Figure 4 there is a qualitative and quantitative difference between non-specific binding to the two surfaces with a much reduced binding to the GLISS surface. The almost negligible amount of material retained at the GLISS surface compared to the classical SAM surface is even more evident from the SPR curves derived from the images.

The available crystallographic structure of GAD65 [6] allowed the selection of a suitable method of coupling of the protein to the chip. Specifically, coupling using surface-exposed cysteine residues in the N-terminal domains allowed the molecule to be immobilised such that the putative antibody epitopes in the C-terminal and PLP domains are exposed and accessible by an antibody molecule (Figure 1). The dimeric architecture of GAD65 presents a challenge for immobilization, since the 2-fold symmetry may place equivalent residues in each monomer on opposite sides of the dimer. There are only two cysteine residues that are on or near the protein surface (Cys101 and Cys304). Cys304 is relatively buried, having only 22 Å^2^ solvent accessible surface area. In addition, its sidechain points inwards towards the body of the protein. Coupling would thus require a significant structural reorganisation, which is highly unlikely. Furthermore, Cys304 residues (from each monomer) are on opposite faces of the dimer, precluding coupling at both sites simultaneously without massive structural reorganisation. Although we cannot discount some coupling at only one Cys304 residue at a time (e.g., “half occupancy”), its low exposure, stereochemistry and location make it a highly unlikely candidate for coupling to the linker. Conversely, Cys101 is highly exposed on the surface of the protein (140 Å^2^ solvent accessible surface area). It is located in the N-terminal domain very close (within 0.15 nm) to its “symmetry-mate” in the dimer. The high exposure and location of Cys101, therefore, make it a highly likely candidate for linker coupling. The high probability of this mode of coupling now allows us to confidently pursue the immobilization of several GAD65 mutants on a single chip. For example we envisage that engineering epitopes on GAD65 may pave the way for improved diagnostic tests for type 1 diabetes using a panel of antibodies as well as human sera. The ability to couple multiple GAD65 proteins on the same chip and measure antibody binding in a reproducible and rapid fashion may ultimately lead to a novel SPRi-based diagnostic immunoassay for type 1 diabetes.

We clearly advocate the use of engineering surface exposed cysteines for immobilisation but recognize that this may not always be possible. A number of alternatives are available, and generally in SPR, coupling using amines, for the most part through accessible lysines, is advocated. It must be noted however, that this generally results in a reduced degree of activity of the immobilised target that may render quantitative analysis difficult. The GLISS surfaces used here may easily be functionalised with carboxyl, thiol or amine groups thus permitting a wide range of immobilisation techniques. However we would like to stress that although the expedient of engineering solvent accessible thiols is somewhat limiting it does optimise accessibility and that whilst this may restrict general applicability we strongly suggest that immobilisation strategies that aim at increasing accessibility and orientation be elaborated rather than blind immobilisation through solvent accessible amines for example. Alternatively one can immobilise on the GLISS surfaces, specific antibodies or haptens either through accessible cysteines or via other coupling techniques, that then allow mild capture of the target molecules.

Although the purpose of the present study was not to explore the detection levels of the SPRi technique we could detect and quantify GAD65-anti-GAD65 interactions between approximately 10^10^ molecules of target GAD65 on the surface and antibody at 4nM concentration in solution. Our limit of detection as discerned from the signal to noise ratio suggests that we can detect a change of approximately 0.01% reflectivity that corresponds to levels of detection of around 30 to 40 ng/ml of protein in solution.

We have characterised the binding of wild type GAD65 to the GAD1 monoclonal antibody using SPRi. Our data illustrate that we have effectively eliminated non-specific interactions with the surface containing the immobilised GAD65 molecules. The implications of this are far reaching; in short not only does this approach obviate the dubious process of background subtraction but gives access to more accurate kinetic and equilibrium values that tend towards more affine measurements since any multiphase behaviour can be separated from non-specific binding. On a broader level, an enhanced signal to noise ratio increases not only the sensitivity but also confidence in the use of SPR to generate kinetic constants that may then be inserted into van't Hoff type analyses to provide comparative ΔG, ΔS and ΔH values, making this an efficient, rapid and competitive alternative to ITC measurements used in drug and macromolecular-interaction mechanistic studies. Finally, and this is particularly evident here, the accuracy of the measurements allows the application of more intricate interaction models than simple Langmuir monophasic binding. The observation that monoclonal antibodies can use multiple binding modes is intriguing. We are currently applying the technology developed here to analyse monoclonal antibody binding to microarrays containing selected mutants of GAD65 (Buckle *et al.* in preparation). The implications are that intramolecular rearrangements associated with antibody binding may be involved.

### Conclusions

The detection and measurement of antibody binding by the type 1 diabetes autoantigen GAD65 represents an example of an antibody-antigen interaction where good structural, mechanistic and immunological data are available. Using SPRi we were able to characterise the kinetics of the interaction in greater detail than ELISA/RIA methods. Furthermore, our data indicate that SPRi is well suited to a multiplexed immunoassay using GAD65 proteins, and may be applicable to other biomarkers.
